# Carbonate Acidizing: Effect of Additives on Reactivity and Wormhole Development

**DOI:** 10.1021/acsomega.6c04459

**Published:** 2026-08-13

**Authors:** Myllena Rosana de Araújo Medeiros, Guilherme Mentges Arruda, Ernani Dias da Silva Filho, José Antonio Barbosa, Leonardo José do Nascimento Guimarães, Mateus Palharini Schwalbert, Marcos Allyson Felipe Rodrigues, José Luis Cardozo Fonseca

**Affiliations:** † Institute of Chemistry, 28123 Federal University of Rio Grande do Norte, Natal, Rio Grande do Norte 59078-970, Brazil; ‡ Department of Chemical Engineering, Federal University of Rio Grande do Norte, Natal, Rio Grande do Norte 59072-970, Brazil; § Department of Geology, 28116 Federal University of Pernambuco, Recife, Pernambuco 50740-530, Brazil; ∥ Civil Engineering Post-Graduation Program, 28116 Federal University of Pernambuco, Recife, Pernambuco 50740-530, Brazil; ⊥ 42506 Petróleo Brasileiro S.A − PETROBRAS, Rio de Janeiro, Rio de Janeiro 21941-915, Brazil; # Department of Petroleum Engineering, Federal University of Rio Grande do Norte, Natal, Rio Grande do Norte 59064-970, Brazil

## Abstract

The reduction in productivity in carbonate reservoirs is often associated with near-wellbore formation damage, making matrix acidizing an essential strategy to restore permeability. In this context, treatment efficiency depends on the balance between reaction kinetics and mass transport, which governs the formation of dominant wormholes. Although additives such as corrosion inhibitors and emulsion preventers are widely used for operational purposes, their effects on the reactive behavior of acid systems remain poorly understood. This study investigates the influence of commercial additives on HCl-based acid systems, using an additive-free system (NA) as a reference. An emulsion preventer (EP), a corrosion inhibitor (CI), and a formulation containing both additives (BA) were evaluated. The EP and CI were used at concentrations of 0.2 and 0.5 vol %, respectively, and the BA formulation contained both additives at the same concentrations. The formulations were evaluated through physicochemical characterization, carbonate dissolution kinetics, and reactive-flow experiments performed on Indiana Limestone samples (18.4% average porosity and 123 mD average permeability) during the injection of 15 wt % HCl until breakthrough. The presence of additives led to a significant reduction in surface tension, suggesting changes in interfacial conditions. Batch reactor experiments showed a pronounced retardation in dissolution kinetics, with reaction times increasing by approximately 4.5-fold, 30.6-fold, and 18.2-fold for the EP, CI, and BA systems, respectively, compared with NA. In reactive-flow experiments, the minimum pore volume to breakthrough (PV_bt_) was reduced from 0.40 PV for the additive-free system (NA) to 0.30, 0.21, and 0.34 PV for the EP, CI, and BA systems, respectively, indicating lower acid consumption to achieve breakthrough under the evaluated laboratory conditions, with the most pronounced effect observed for the CI system. Microcomputed tomography analysis revealed that acid systems containing additives promoted the formation of dominant wormholes at lower interstitial velocities, shifting the optimal acidizing condition toward lower injection rates. Under the evaluated laboratory conditions, this behavior is likely associated with reaction retardation induced by the additives, which may reduce premature acid consumption and enable deeper wormhole propagation before significant radial enlargement occurs. The results demonstrate that acid formulation plays a key role in controlling the dissolution regime and wormhole development. Even at low concentrations, the commercial additives modified dissolution kinetics, reduced the pore volume required to achieve breakthrough, and altered wormhole development, shifting the optimum acidizing condition toward lower injection rates under the evaluated laboratory conditions. These findings demonstrate that commercial acidizing additives should be considered as active formulation components capable of modifying dissolution kinetics, acid consumption, and wormhole development under reactive-flow conditions.

## Graphical abstract

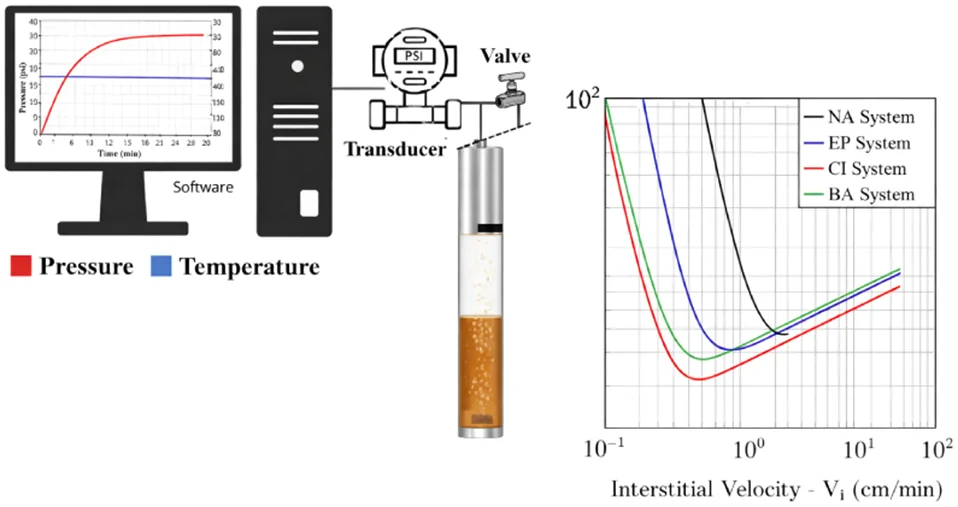

## Introduction

Petroleum reservoirs are located in geological formations composed of porous rocks where the storage capacity of fluids is described by the fraction of the total rock volume occupied by void space (porosity, Φ).^1,2^ However, porosity by itself does not ensure that fluids can flow effectively through the rock. Fluid transport depends on the degree of connectivity between these pores, which governs the permeability and controls the ability of oil, water, or gas to flow through the porous medium.^3,4^

Even formations that initially exhibit well-connected pore networks may experience significant reductions in permeability as a result of alterations caused by drilling, completion, or production operations.^5^ These changes, also known as formation damage, become more critical in the near-wellbore region (NWR), where converging flow increases the impact of any restriction to fluid movement.^6^ The formation damage leads to decreased productivity in producing wells or reduced injectivity in injection wells. In such cases, stimulation techniques are employed to restore or enhance the flow capacity of this region, enhancing operational efficiency.^7^

Matrix acidizing in carbonate formations is designed to enhance near-wellbore permeability by injecting an acidic solution capable of dissolving the rock matrix and restoring effective flow pathways.^8^ It is called a matrix treatment because the acid is injected at pressures below the formation fracture. When hydrochloric acid comes into contact with carbonate minerals, such as calcite or dolomite, it reacts with the rock matrix, producing soluble salts and CO_2_, dissolving the rock matrix and generating highly conductive flow channels (wormholes).^9^

The objective is to recover, or even improve, the permeability in the near-wellbore region rather than to affect a large portion of the reservoir. The development of these channels is governed by the balance between overall rate of dissolution and the rate of transport by convection. When this balance approaches the favorable regime, dominant pattern wormholes are formed.^10^ This pattern is considered advantageous due to its deep penetration and reduced acid consumption.^11^ As a result, the treatment becomes more efficient, as the greater reduction in the skin effect improves well productivity, thereby enhancing the economic return of the operation.^12^

Several additives must be used in acid formulations to support the execution of carbonate matrix treatments.^13,14^ In these acid systems, corrosion inhibitors play the primary role of protecting tubulars and downhole equipment from the aggressive environment created by different acids, such as HCl, formic acid, and acetic acid. This protection commonly occurs through the formation of a temporary film that slows metal dissolution.^15,16^ This protective mechanism becomes even more critical when strong acids are used or when treatments are carried out in high temperature wells.^17^

On the other hand, emulsion preventers are added to mitigate the formation of stable emulsions between acid, brine, crude oil, and fines released during the acidizing.^18,19^ These emulsions can not only obstruct pore channels, causing new formation damage, but also hinder fluid cleanup, limiting the treatment efficiency.

Although each additive is introduced for a specific purpose, their presence can affect other parts of the acid–rock interaction.^20^ By changing viscosity, interfacial behavior, wettability, or even modifying how reactants and products move through the porous media, these components can impact mass transfer, overall dissolution kinetics, and the wormhole patterns. Thus, recognizing these additional effects is important to understand how each or both additives affects the carbonate acidizing treatment.^21^

Previous studies have demonstrated that variables such as acid concentration,^22^ mineralogy,^23^ temperature^24,25^ and injection rate^11^ play a key role in controlling dissolution regimes and wormhole formation. However, although corrosion inhibitors and emulsion preventers are essential components of carbonate acidizing formulations, their effects have been investigated primarily in terms of corrosion protection, emulsion prevention, and fluid compatibility.^15,26–28^

Nevertheless, a relevant knowledge gap remains regarding how these additives influence acid–rock interactions during carbonate matrix acidizing. In particular, their effects on acid–rock reactivity, acid consumption, PV_bt_ behavior, and wormhole development under representative reservoir acidizing conditions, including elevated temperatures, have received limited attention in the literature, despite their widespread use in field formulations.^21,29^

It is important to emphasize that, in this manuscript, the terms corrosion inhibitor and emulsion preventer refer to commercial additives according to their intended field functions. The present study did not directly evaluate corrosion inhibition efficiency or emulsion prevention performance. Instead, these additives were investigated as components of HCl-based acid formulations to assess their influence on carbonate dissolution and reactive-flow behavior.

Therefore, this study investigates the effects of two commercial acidizing additives—a corrosion inhibitor and an emulsion preventer—evaluated individually and in combination, on carbonate dissolution and reactive-flow behavior during matrix acidizing. By combining physicochemical characterization, batch-reactor dissolution tests, core-flooding experiments, PV_bt_ curve analysis, Buijse–Glasbergen model fitting, inlet-face inspection, and microcomputed tomography, this work provides a direct assessment of how commercial acidizing additives influence carbonate reactivity, acid utilization, and wormhole development.

## Materials and Methods

The experimental procedures adopted in this study were designed to evaluate how commercial additives (a corrosion inhibitor and an emulsion preventer), applied in field acidizing operations, affect the properties of the stimulation fluid and the acidizing process. This section describes the materials used and the methodologies used for carbonate and acid characterization, dissolution rate analysis of acid systems, and reactive flow experiments in porous media.

### Materials

The materials used in this study include the carbonate core samples, KCl brine and the acid formulations prepared for the experiments. The samples were selected based on their mineralogical composition and petrophysical properties. The KCl solution was used to saturate the cores, assess their permeability, and remove unreacted acid after breakthrough.

The stimulation fluids were prepared using a baseline HCl solution, to which the corrosion inhibitor, the emulsion preventer, or both additives were incorporated, depending on the formulation being evaluated. Because of confidentiality agreements with the supplier, the exact chemical composition of these commercial additives cannot be disclosed. However, to improve reproducibility and facilitate comparison with future studies, additional nonproprietary information available from the technical and safety data sheets is provided. The corrosion inhibitor is a surfactant-based commercial additive mainly composed of cationic surface-active compounds, including quaternary ammonium salts, alcohols, and aldehyde-containing components. According to the supplier information, it is supplied as a brown liquid, with a density/specific gravity of 0.988 g/cm^3^ and a pH of 3.25. The emulsion preventer is a nonionic surfactant-based additive containing ethanol as the solvent. According to the supplier information, it is supplied as a colorless liquid, with a density/specific gravity of 1.001 g/cm^3^ and a pH of 6.60. These pH values refer to the neat commercial additives. The active-content range and supplier-recommended dosage range are not reported due to supplier confidentiality. The concentrations adopted in this study, 0.5 vol % for the corrosion inhibitor and 0.2 vol % for the emulsion preventer, were selected based on the field-practice formulation used for carbonate acidizing operations and were applied to evaluate the individual and combined effects of these additives on acid–rock reactivity and wormhole development.

#### Carbonate Samples

For the experimental tests, carbonate rock cores were acquired from Kocurek Industries, Inc. The samples used in this study consist of Indiana Limestone cylinders with a diameter of 1.5 in. (3.81 cm) and a length of 6 in. (15.24 cm).

Indiana Limestone was selected because this work focuses on evaluating the effect of additives, and this carbonate is extensively used in the literature owing to its predominantly calcitic composition, compressive strength compatible with the confining pressures applied in the core flooding experiments, and low heterogeneity compared with other carbonate rocks. In addition, its broad permeability range facilitates the selection of samples with petrophysical properties appropriate for reactive-flow studies.

Several authors have employed this rock type to evaluate the effects of operational parameters and stimulation fluid composition on matrix acidizing performance.^4,13,30^

#### Injection Fluids: KCl Brine and Acid Formulations

The brine used in the experiments consisted of a 4 m/v% potassium chloride (KCl) solution, prepared with distilled water and analytical-grade KCl supplied by Synth, Brazil. This solution was used to saturate the samples prior to testing, determine permeability during flow, and remove any remaining acid after acidizing.

To evaluate the effect of the additives, four solutions were formulated: hydrochloric acid without additives (NA), hydrochloric acid with an emulsion preventer (EP), hydrochloric acid with a corrosion inhibitor (CI), and hydrochloric acid with both the emulsion preventer and the corrosion inhibitor (BA). The NA solution is the reference system. The EP and CI solutions were prepared to assess the individual effect of each additive. The fourth solution (BA) has the same composition as the formulation used in field operations. It was prepared to evaluate the combined effects of both additives.

All acid systems were prepared in the same order of addition: distilled water, corrosion inhibitor, emulsion preventer, and 37 wt % HCl. For formulations that did not contain one or more of these additives, the preparation order was maintained by omitting the absent components. The order of addition was adopted to reproduce the standard preparation procedure used for these acidizing formulations. Concentrated HCl was added as the final component to minimize direct contact between the acid and the undiluted additives during mixing, reducing the likelihood of local overheating, precipitation, or phase instability. The final composition of each acid system is presented in Table 1.

**Table 1:** Composition of the Acid Systems Used in the Experiments (NA, EP, CI, and BA)

	Composition (% v/v)		
Acid System	HCl 15 wt %	Emulsion Preventer	Corrosion Inhibitor
NA^a^	100.0	0.0	0.0
EP^b^	99.8	0.2	0.0
CI^c^	99.5	0.0	0.5
BA^d^	99.3	0.2	0.5

a No additives.

b Emulsion preventer added.

c Corrosion inhibitor added.

d Both additives included.

The additive concentrations used in this study were selected based on the field-practice formulation adopted for carbonate acidizing operations. The emulsion preventer and corrosion inhibitor were used at concentrations of 0.2 and 0.5 vol %, respectively. These concentrations were kept constant in the individual additive systems and in the combined formulation to allow direct comparison between the isolated and combined effects of each additive on acid–rock reactivity, PV_bt_ behavior, and wormhole development.

### Characterization of the Carbonate Samples

To reduce variability in the acid stimulation experiments and improve the understanding of the effects attributed to the acid formulations and evaluated additives, the carbonate rock samples were previously characterized. The characterization included the determination of fundamental petrophysical properties such as permeability and porosity, as well as mineralogical composition analysis.

#### Petrophysical Analysis

Each core sample was measured and weighed before and after brine saturation, and porosity was calculated from the difference between the dry and saturated masses. The permeability was obtained from the brine flow through the core during the core flooding experiments. Additional details on the petrophysical characterization of the rocks are provided in the sections “Brine Saturation” and “Liquid Permeability Measurement”.

#### Compositional Determination

The chemical and mineralogical composition of the samples was determined by X-ray diffraction (XRD) and X-ray fluorescence (XRF). The XRF analysis was performed using a Shimadzu EDX-720 energy-dispersive X-ray fluorescence (EDXRF) spectrometer. The XRD analyses were carried out on a Bruker D2 Phaser diffractometer equipped with a LynxEye detector, operating at room temperature (25 °C) and using Cu Kα radiation (λ = 1.54 Å). Measurements were conducted over the 10°–80° (2θ) range, with a step size of 0.02° and a scanning rate of 2°/min.

### Characterization of the Acid Formulations

The physicochemical properties of the acid solution directly affect fluid flow through the porous medium, rock–fluid interactions, and, consequently, the performance of the solution during acid stimulation. Therefore, the density, viscosity, and surface tension of the acid systems were determined to assess the impact of the additives on the core flooding results.

#### Density

The density of the acid formulations was measured using an Anton Paar digital densimeter, model DMA 4500 M, operating at 60 °C. The solutions were injected into the measurement cell, which had been previously calibrated with distilled water, following the procedure recommended by the manufacturer. Two aliquots of each acid system were analyzed to determine the average value and the standard deviation.

#### Dynamic Viscosity

The dynamic viscosity of the fluids was determined using a Cannon–Fenske viscometer No. 100 immersed in an ultrathermostatic bath at the target temperature of 60 °C. During each measurement, the flow time between the two defined marks was recorded. Each solution was introduced into the capillary, previously cleaned and leveled, and the efflux time was measured using a digital stopwatch. The measurement was repeated in triplicate to ensure the precision of the results. The dynamic viscosities were then calculated using the eq 1.

1 $$\mu_{s}=\mu_{w}\frac{\rho_{s}t_{s}}{\rho_{w}t_{w}}$$

where μ_s_, ρ_s_, and t_s_ refer, respectively, to the viscosity, density, and efflux time of the acid solution, while μ_w_, ρ_w_, and t_w_ denote the same parameters measured for distilled water.

#### Surface Tension

The surface tension of the acid formulations was determined using a K20 Force Tensiometer (KRÜSS GmbH) employing the Du Noüy ring method. The ring was cleaned before each measurement to prevent contamination, and the solutions were then placed in the instrument’s sample vessel for analysis.

The equipment accounts for the solution density and applies air-density corrections to ensure accurate results. For each formulation, two independent aliquots were prepared, and one measurement per aliquot was performed. The analyses were conducted at 25 °C due to laboratory limitations.

### Dissolution Rate Analysis of Carbonate Rock

Understanding the dissolution kinetics of each acid system can help interpret the behavior observed in core flooding experiments and provide insight into the mechanisms governing wormhole development. By quantifying reaction rates, the effect of additives on acid–rock interactions can be evaluated and correlated with acid consumption efficiency in matrix stimulation processes.

To determine the reaction kinetics between the carbonate rock and the different stimulation fluids, batch reactor experiments were performed using a setup capable of continuously monitoring the pressure variation generated during dissolution.^29^ The tests were conducted with Indiana Limestone samples and the various acid formulations (NA, EP, CI, and BA), allowing the evaluation of the impact of the additives. All experiments were carried out at 60 °C and performed in duplicate to obtain average reaction curves and ensure reproducibility.

Semidisk samples were prepared by first cutting the original Indiana Limestone cores into disks and then splitting each disk in half. Each piece was sanded to a final mass of approximately 2 g, then labeled and stored until testing.

As shown in Figure 1, the kinetic tests were carried out in a closed batch reactor equipped with a 0.345 MPa (50 psi) pressure transducer, a relief valve, and a data acquisition system programmed to record internal pressure every second. The reactor volume was adjusted to ensure that the maximum pressure during dissolution did not exceed 0.28 MPa (40 psi).

**Figure 1:**
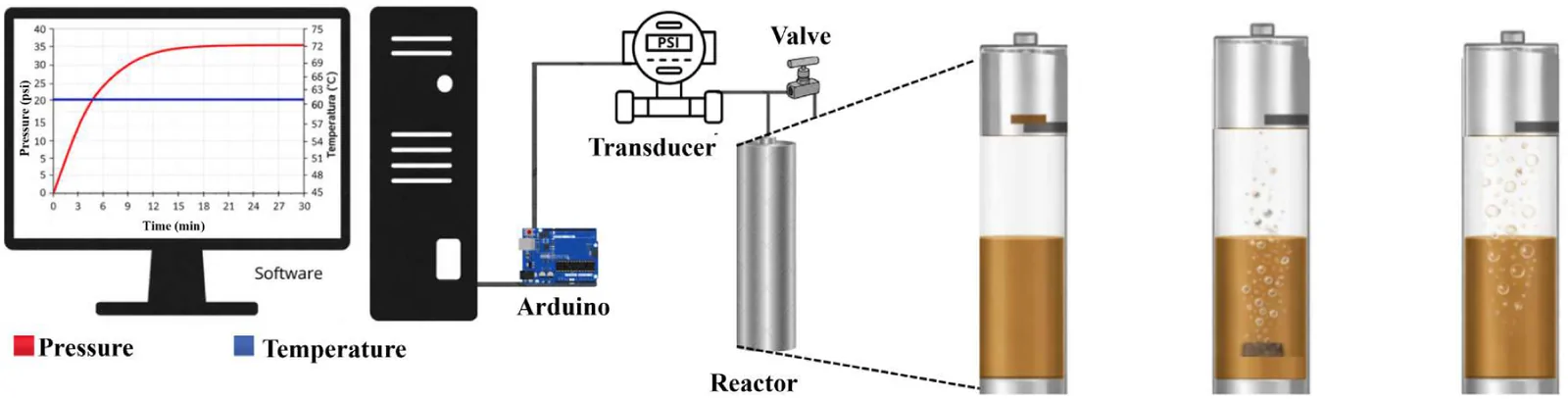
Illustration of the reactor used in the experimental tests.

Approximately 100 mL of the acid solution was added, maintaining an excess of reagent, and the prepared sample was placed on an internal support.

Once the closed reactor system was sealed, the reaction was initiated by mechanical agitation, which caused the carbonate sample to fall from the internal support into the acid solution. The reaction was monitored continuously, and progress was tracked by the pressure increase resulting from CO_2_ release. In this study, the complete dissolution time was operationally defined as the elapsed time from the onset of the pressure increase to the point at which the pressure–time curve reached a plateau, indicating that CO_2_ generation had ceased due to complete consumption of the carbonate sample. The collected data were then used to construct dissolution curves over time.

### Core Flooding Experiments

The methodology was organized into successive stages. First, the samples were selected and saturated with a 4 m/v% KCl solution. Permeability measurements were obtained during the flow experiment, using the core flooding system itself, in a step performed prior to acid injection.

After the acid flow, a set of posttest activities was carried out, including visual evaluation of the sample faces, calculation of the pore volume to breakthrough (PV_bt_), fitting of the experimental data to the Buijse–Glasbergen model, and microcomputed tomography (μCT). Further details are provided in the following subsections.

#### Brine Saturation

Initially, each core was measured and weighed to determine its geometric volume and dry mass. The samples were then fully saturated using an automatic saturator (Vinci Technologies). To ensure a higher degree of saturation, the device first applies a vacuum and subsequently a pressure of 6.894 MPa (1000 psi). The cores are kept pressurized for 2 h. After this step, the samples were weighed and stored submerged in 4 m/v% KCl until the setup of their respective core flooding experiments. The effective porosity was calculated from the difference between the dry and saturated masses, considering the sample dimensions and the brine density.

#### Liquid Permeability Measurement

The core flooding experiments were conducted under controlled pressure and temperature conditions. The pore pressure during the experiment was maintained at 8.27 MPa (1200 psi), while the confining pressure applied to the core holder was set to 13.79 MPa (2000 psi). The system temperature was stabilized at 60 °C using an external heating oven.

Brine was injected at a constant flow rate of 2 mL/min to determine the liquid permeability of all samples. This step also ensured system tightness verification and full saturation of the samples. Injection was continued until steady-state flow conditions were achieved, defined as stabilization of the pressure differential after the injection of at least five pore volumes (≥5 PV). The permeability (k) was then calculated using Darcy’s law for incompressible single-phase flow (eq 2).

2 $$k=\frac{q\mu L}{A\Delta p}$$

For this calculation, the injection flow rate (q), the core length and cross-sectional area (L and A, respectively), the pressure difference between inlet and outlet (Δp), and the brine viscosity (μ) were considered. The pressure difference was measured using a differential pressure transducer (YOKOGAWA EJA100A, Japan), with a measurement span 8.62 × 10^–4^ to 8.62 × 10^–2^ MPa (0.125–12.5 psi) and a reference accuracy of ±0.065% of the calibrated span. The μ values were obtained from the viscosity tables for aqueous KCl solutions.^31^

#### Carbonate Matrix Acidizing

After the permeability measurement, the operational configuration was kept unchanged for the acid injection stage. The acid line, which had been filled and pressurized together with the brine line at the beginning of the experiment, was already at the system pressure, preventing any backflow of brine into the accumulator during the fluid switch through the automatic valves.

Before each acid injection, the brine flow rate was adjusted to match the planned acid injection rate, and the system was allowed to reach steady conditions. Once equilibrium was achieved, the brine valve was closed and the acid valve was opened simultaneously, ensuring a smooth transition between fluids.

Breakthrough was identified by a sudden reduction in the pressure differential along the core, with Δ*p* approaching near-zero values relative to the initial condition, together with the change in the effluent pH, which was continuously monitored during the experiment. Acid injection was stopped immediately after breakthrough to limit further carbonate dissolution. The sample was then subjected to a KCl brine postflush at 10 mL/min to remove unreacted acid from the pore space and minimize continued acid–rock reaction before posttest analyses, including inlet-face inspection and microcomputed tomography.

All pressure, temperature, and pump operation data were automatically recorded by a data acquisition system, with measurements collected at a frequency of one data point per second.

#### PV_bt_ Curve Analysis

The PV_bt_ curve was constructed following a methodology reported in the literature for carbonate acidizing under core flooding conditions, based on a series of core flooding experiments conducted at different flow rates.^12,21^ The influence of injection rate and interstitial velocity on the amount of acid required to reach breakthrough was evaluated in terms of pore volumes.

For each experiment, the PV_bt_ value was obtained as the ratio between the volume of acid injected to breakthrough (V_inj_) and the pore volume of the sample (PV), as shown in eq 3.

3 $${\mathrm{PV}}_{\mathrm{bt}}=\frac{V_{\mathrm{inj}}}{\mathrm{PV}}$$

The PV_bt_ curves presented in the Results section are shown as a function of flow rate and interstitial velocity, with the latter used as the standard parameter for the subsequent analyses. The interstitial velocity (V_i_, cm/min) was calculated from the injection rate (q, cm^3^/min), the sample cross-sectional area (A, cm^2^), and the effective porosity (Φ, −), as given in eq 4.

4 $$V_{i}=\frac{q}{A\times \Phi }$$

The PV_bt_ behavior exhibits three characteristic trends: high PV_bt_ at low interstitial velocities, a minimum PV_bt_ region at intermediate velocities, and increasing PV_bt_ at high interstitial velocities. For each formulation, injection rates were selected to resolve these trends across the full curve. Experimental points were considered consistent when they followed the expected PV_bt_ profile and contributed to the definition of the characteristic curve behavior.

#### Buijse–Glasbergen Model Fitting

According to the model proposed by Buijse and Glasbergen, the PV_bt_ (previously described in eq 3) can be further adapted to eq 5. The resulting expression relates the PV_bt_ to the interstitial acid velocity (V_i_), the wormhole opening efficiency factor (W_eff_), and the wormhole growth factor (W_B_). The W_eff_ and W_B_ parameters are constants that can be obtained by fitting eq 5 to the experimental data.

5 $${\mathrm{PV}}_{\mathrm{bt}}=\frac{V_{i}^{1/3}}{W_{\mathrm{eff}}\times {\left(1-e^{-W_{B}\times V_{i}^{2}}\right)}^{2}}$$

#### Rock Surface Analysis after Acid Flow

The inlet face of the samples is the first region of interaction between the acid and the porous medium. It is particularly sensitive to variations in fluid formulation and experimental conditions. Although large mineralogical variations are not expected along the samples due to their high calcite content, differences in the reactive behavior of the acid systems and in the injection conditions can modify the intensity of surface dissolution at this interface. Therefore, standardized photographic documentation of the inlet face was carried out to provide a visual reference, to support the interpretation of the initial dissolution mechanisms, and to complement the subsequent analyses.

#### Microcomputed Tomography Analysis

The tomographic images of the samples were acquired using a Nikon XT H 225 ST system fitted with a tungsten X-ray source. Each full scan required roughly 25 min, with individual projections captured at 500 ms exposure. Image acquisition was conducted under the following conditions: 110 μA beam current, 180 kV accelerating voltage, and a 0.5 mm aluminum filter. All data sets were generated with a spatial resolution of 60 μm per pixel.

Following the acquisition stage, the projections were converted into three-dimensional volumes using CT Pro 3D. Subsequent processing was performed in VG Studio Max 3.4.4, where the orientation of each core was realigned to achieve the highest possible orthogonality with respect to the bottom face of the core sample. From each reconstructed volume, a cylindrical subregion was selected, maximizing its dimensions while accounting for geometric imperfections present in the samples. A median filter was applied to reduce image noise and improve not only the segmentation process, but also the subsequent digital petrophysical evaluations.

Segmentation was then performed to separate the pore network from the solid matrix, providing valuable information on the spatial distribution and morphology of the wormholes formed under the different acid solutions and injection conditions.

## Results and Discussion

### Characterization of the Carbonate Samples

The mineralogical and chemical composition of the Indiana Limestone samples was evaluated by X-ray diffraction (XRD) and X-ray fluorescence (XRF) to verify the compositional comparability of the carbonate samples used in the acidizing experiments.

The XRD pattern of the Indiana Limestone sample is presented in Figure 2. The diffractogram shows well-defined reflections characteristic of calcite, indicating that calcite is the dominant crystalline phase in the rock. The main peaks were observed at 2θ values of 23.06°, 29.46°, 36.00°, 39.44°, 43.20°, 47.50°, 48.58°, and 57.46°, corresponding to the (012), (104), (110), (113), (202), (018), (116), and (122) planes of calcite, respectively.^21,29^ No additional reflections associated with secondary crystalline phases were detected within the technique’s resolution limits. This result indicates that the sample is essentially monomineralic, which is advantageous for isolating the effects of the acid formulations and additives on the acidizing response.

**Figure 2:**
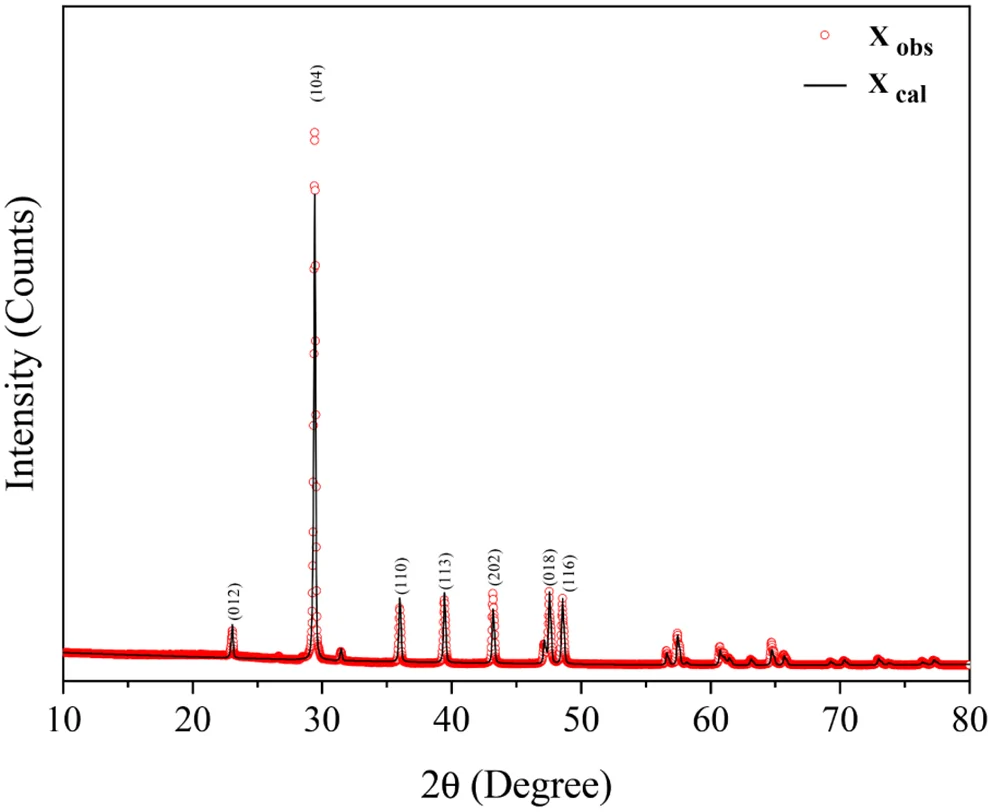
X-ray diffraction pattern of the carbonate rock sample. Open red circles indicate the observed intensities (*X*_obs_), while the black solid line represents the calculated profile through Rietveld refinement (*X*_cal_).

The XRF analysis corroborates the XRD results by showing a predominance of CaO in the bulk chemical composition. The measured CaO content was 96.05 ± 1.86 wt %, while the remaining oxides were present only in trace amounts, including SiO_2_, Fe_2_O_3_, Al_2_O_3_, and SrO. Although XRF does not directly identify mineral phases, this high CaO content is fully consistent with a carbonate rock composed predominantly of calcium carbonate.

The combined XRD and XRF results confirm that the Indiana Limestone used in this study is a highly calcitic material with negligible contribution from accessory minerals. This high mineralogical purity minimizes uncertainties associated with secondary acid-consuming phases, allowing the observed differences in dissolution behavior to be attributed primarily to the characteristics of the acid systems rather than to rock mineralogy.

### Characterization of the Acid Formulations

The physicochemical characterization of the acid formulations was performed to assess the effects of additives commonly used in acid stimulation fluids, considering their individual and combined contributions. The experimental results for density, viscosity, and surface tension of the four evaluated formulations are summarized in Table 2.

**Table 2:** Characterization of the Acid Systems Used in the Experiments

	Physicochemical Properties		
Acid System	Density—60 °C (g·cm^–3^)	Viscosity—60 °C (mPa·s)	Surface Tension—25 °C (mN/m)
NA^a^	1.0563 ± 0.0002	0.648 ± 0.007	60.51 ± 1.82
EP^b^	1.0556 ± 0.0001	0.656 ± 0.010	40.49 ± 0.29
CI^c^	1.0556 ± 0.0001	0.678 ± 0.012	31.17 ± 0.11
BA^d^	1.0559 ± 0.0001	0.647 ± 0.013	31.47 ± 0.12

a No additives.

b Emulsion preventer added.

c Corrosion inhibitor added.

d Both additives included.

The measured density values indicate that the presence of additives did not lead to significant changes in this property when compared to the reference acid fluid, suggesting that the additive concentrations employed are not sufficient to meaningfully alter the density of the system. As a result, the evaluated formulations do not significantly modify fluid density and, therefore, are not expected to affect the hydrostatic pressure during operation.

These results are consistent with literature data for 15 wt % HCl solutions, whose density values typically fall within the range of 1.07–1.08 g·cm^–3^ at 25 °C and decrease progressively with increasing temperature.^29,32^

Regarding dynamic viscosity, the formulations showed very similar values at 60 °C (0.647–0.678 mPa·s), indicating that the additives had only a minor effect under these conditions. The maximum increase was approximately 0.03 mPa·s (CI formulation), suggesting a small increase in flow resistance and, in principle, a limited reduction in H^+^ effective diffusivity during the reaction. In addition, the measured values are consistent with those reported in the literature for 15 wt % HCl solutions.^33^

The surface tension results show that the presence of the additives (whether EP, CI, or their combination) was associated with a reduction in the acid fluid surface tension compared with the reference solution. This behavior is consistent with the presence of surfactants in these formulations, whose amphiphilic structures (polar groups and hydrophobic chains) preferentially adsorb at the liquid–gas interface and reduce the system’s interfacial energy, as widely described for surfactants in acidizing fluids.^34^

In addition, other constituents commonly present in these additive packages, such as alcohols, may lower surface tension by reducing intermolecular cohesion in the aqueous phase and by preferentially partitioning to the liquid–gas interface.^35^ Lower surface tension is beneficial for acidizing because it reduces capillary trapping, facilitating flow into small pore throats and spent-acid recovery.

### Dissolution Rate Analysis of Carbonate Rock

Figure 3 presents the results of batch reactor tests comparing the impact of each additive in the acid formulation by evaluating dissolution kinetics and the total dissolution time of the carbonate sample. In this context, tests were conducted with 15 wt % HCl without additives (NA), 15 wt % HCl with emulsion preventer (EP), 15 wt % HCl with corrosion inhibitor (CI), and with both additives (BA) at 60 °C.

**Figure 3:**
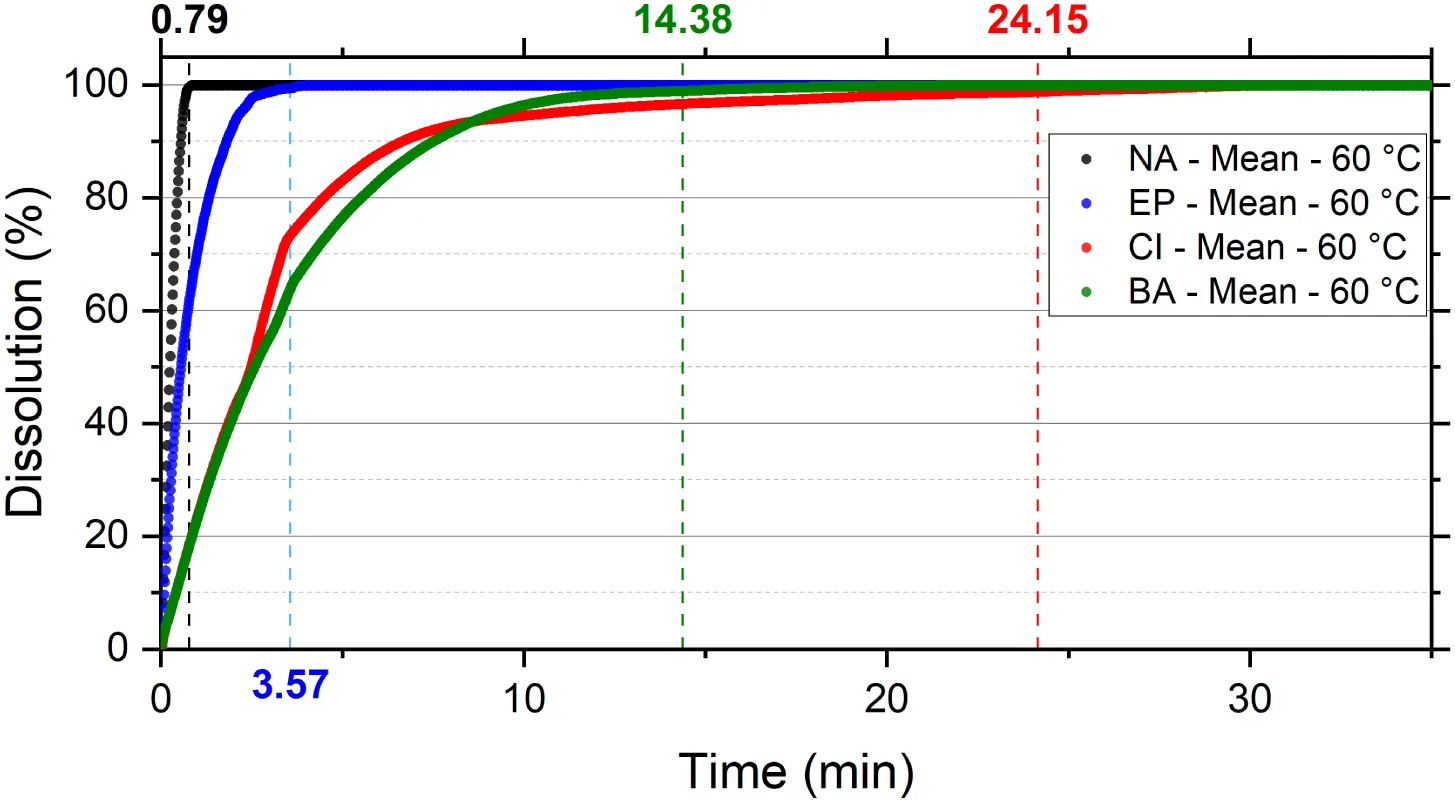
Dissolution kinetics curves obtained from batch reactor experiments at 60 °C for different acid systems (NA, EP, CI, and BA).

At 60 °C, the presence of additives significantly increases the time required for complete carbonate dissolution. The additive-free acid (NA) exhibited the shortest dissolution time (0.79 min), whereas EP and CI increased this time by approximately 4.5 and 30.7 times, respectively. The BA formulation showed an intermediate behavior, with an increase of approximately 18.2 times relative to NA, indicating that combined additives modify reaction kinetics in a nonlinear manner.

Calcite exhibits high reactivity with HCl, and under typical reservoir conditions dissolution is often mass-transfer limited.^36,37^ In this context, the dissolution time results suggest that the presence of additives may modify mass-transfer behavior. Adsorption of organic species can modify interfacial properties and reduce the accessibility of the reactive carbonate surface.^38^ In parallel, foam formation and stabilization during CO_2_ release introduce a dispersed gas phase that behaves as a physical barrier, decreasing the effective contact area and increasing diffusional resistance.^33,39^

Consistently, the CI and BA formulations, which contain higher concentrations of surfactant species, exhibit a more delayed initial response compared with the other systems. This behavior is associated with the action of surface-active components, which temporarily reduce mass-transfer efficiency at the reaction–fluid interface, and is supported by the observed reduction in surface tension for both systems (CI and BA) relative to the additive-free acid, reinforcing the role of surfactant-induced interfacial effects during the initial stage of the reaction.^40^

However, despite exhibiting similar surface tension values, CI and BA show distinct temporal behavior after approximately 2.5 min, indicating that surface tension alone does not fully explain the observed differences. Instead, the results suggest that the surfactant composition governs the evolution and stability of gas–liquid structures formed during CO_2_ release, which controls the apparent interfacial resistance during the reaction.

Systems with a higher contribution of ionic surfactants (CI) are expected to favor stronger stabilization of gas–liquid interfaces through electrostatic repulsion between opposing film surfaces, promoting greater foam persistence and reduced bubble coalescence. In contrast, the presence of a larger fraction of surfactants with little or no interfacial charge contribution in BA may reduce electrostatic stabilization by decreasing the density of charged species at the interface. According to foam stability theory, repulsive interactions increase the energy barrier for film rupture, whereas local fluctuations in surfactant concentration can reduce these repulsive forces and facilitate coalescence.^41^ Consequently, differences in surfactant composition may modify the stability of transient gas–liquid structures formed during CO_2_ evolution, which act as a temporary resistance to mass transfer and influence the continuation of carbonate dissolution. This interpretation is proposed as a mechanistic hypothesis based on the pressure-time behavior observed in the batch reactor tests.

As an insight from these results, the increase in dissolution time observed in the presence of additives indicates a more appropriate balance between reaction kinetics and mass transport. This behavior suggests greater acid availability for deeper penetration into the porous medium before complete reaction, which may favor more efficient conditions for wormhole development with a lower total acid volume.^30,42^

### Core Flooding Experiments

The experiments were conducted to evaluate the effect of additives on carbonate acidizing. As previously mentioned, four HCl-based systems were analyzed: EP includes only the emulsion preventer; CI includes only the corrosion inhibitor; BA contains both additives; and NA consists of HCl without additives and serves as our reference system.

A total of 24 experiments were conducted. At least five experimental points from each system were collected to properly capture the PV_bt_ curve behavior. Table 3 summarizes the number of experiments performed, the core sample used, the acid system used, and the results from the petrophysical characterization and the reactive flow tests in porous media. In the Sample Code column, the letters indicate the acid system used (NA, EP, CI, or BA), while the numbers represent the flow rate applied in each experiment.

**Table 3:** Petrophysical Characterization and Experimental Results of the Core Flooding Experiments

Test Number	Sample Code	Acid System	Porosity (%)	Permeability (mD)	q (mL/min)	V_i_ (cm/min)	PV_bt_
1	NA-2.0	NA	18.5	113.86	2.0	0.95	1.51
2	NA-4.0	NA	19.1	112.04	4.0	1.84	0.57
3	NA-8.0	NA	18.9	73.72	8.0	3.71	0.40
4	NA-16.0	NA	15.9	75.19	16.0	8.83	0.40
5	NA-28.0	NA	19.0	78.31	28.0	12.9	0.66
6	EP-0.5	EP	17.8	79.84	0.5	0.25	1.67
7	EP-1.0	EP	18.5	76.70	1.0	0.47	0.85
8	EP-2.0	EP	18.8	129.79	2.0	0.93	0.30
9	EP-4.0	EP	18.3	78.41	4.0	1.91	0.35
10	EP-16.0	EP	18.2	77.89	16.0	7.72	0.57
11	CI-0.3	CI	18.2	131.27	0.3	0.14	1.20
12	CI-0.5	CI	17.5	87.14	0.5	0.25	0.80
13	CI-1.0	CI	18.6	138.90	1.0	0.47	0.21
14	CI-2.0	CI	18.8	141.71	2.0	0.93	0.21
15	CI-4.0	CI	18.3	123.02	4.0	1.92	0.30
16	CI-16.0	CI	17.9	89.82	16.0	7.83	0.54
17	BA-0.3^a^	BA	19.2	99.56	0.3	0.14	2.30
18	BA-0.4	BA	16.9	190.76	0.4	0.21	1.30
19	BA-0.5	BA	17.2	360.72	0.5	0.26	0.39
20	BA-1.0	BA	20.2	147.57	1.0	0.43	0.34
21	BA-2.0	BA	19.9	121.25	2.0	0.88	0.36
22	BA-3.1	BA	20.0	227.03	3.1	1.38	0.35
23	BA-4.0	BA	17.6	167.37	4.0	1.99	0.34
24	BA-16.0	BA	20.0	208.34	16.0	7.03	0.56

a Incomplete experiment. The test did not reach a true breakthrough because intense inlet-face dissolution caused loss of confinement. This point is shown for transparency and completeness, but it was not used to determine the optimum PV_bt_ condition or for Buijse–Glasbergen model fitting.

The flow rates used in the core flooding experiments were selected to cover a broad range of interstitial velocities and to capture the characteristic shape of the PV_bt_ curve for each formulation. This range included low-flow-rate conditions, where acid consumption is expected to be high because of excessive reaction near the inlet region, intermediate conditions associated with dominant wormhole formation and minimum PV_bt_, and high-flow-rate conditions, where inefficient acid utilization may occur because of insufficient acid–rock contact time. For the additive-containing systems, lower flow rates were included to improve the resolution of the PV_bt_ curve and to evaluate a possible shift of the optimum acidizing condition toward lower interstitial velocities.

The interpretation of the PV_bt_ curve is based on the overall behavior of the set of experiments performed for each acid formulation, relating the flow rate and interstitial velocity to the volume of acid required to reach breakthrough. Each PV_bt_ data point was derived from independent core-flooding experiments conducted at different flow rates, as replicate measurements were not possible due to the destructive nature of the acidizing process and the inherent variability in sample petrophysical properties. Despite this limitation, the set of experimental points is consistent with the characteristic behavior of PV_bt_ curves, showing a well-defined trend across different flow rates and the volume of acid required to reach breakthrough. This behavior suggests the representativeness of the data set and allows comparison among the evaluated systems.

The uncertainty associated with PV_bt_ was mainly related to the determination of the injected acid volume up to breakthrough and to the pore volume of each core sample. Since PV_bt_ was calculated as *V*_inj_/PV, uncertainties in PV_bt_ propagate from the acid injection flow rate, the breakthrough time, and the pore-volume calculation. The injected volume was determined from the imposed pump flow rate and the time elapsed until breakthrough, with pressure, temperature, and pump data recorded at 1 s intervals. Therefore, the temporal resolution of breakthrough detection was limited to approximately 1 s. Breakthrough identification was supported by the abrupt reduction in the pressure differential along the core, approaching near-zero values relative to the initial condition, together with the change in effluent pH. The pressure differential was measured using a differential pressure transducer with a reference accuracy of ±0.065% of the calibrated span. Additional uncertainty in PV arises from the measurement of dry and saturated masses, sample dimensions, and brine density used to calculate effective porosity. Permeability uncertainty is associated with the same flow rate, viscosity, sample geometry, and pressure drop terms as in Darcy’s law. Although these sources may affect the absolute PV_bt_ values, they do not alter the comparative interpretation of the PV_bt_ curves, which were based on the overall trend observed for each acid formulation.

It was not possible to perform a prior selection based on petrophysical property ranges, since permeability was only determined after the flow experiments began. Although porosity showed relatively limited variation (15.9–20.2%), permeability ranged from 73.72 to 360.72 mD, reflecting the natural heterogeneity of Indiana Limestone. Most of this dispersion was associated with Sample 19, which exhibited a permeability of 360.72 mD, higher than that of the other BA samples. This difference represents a limitation of the data set because permeability and pore-structure heterogeneity can influence acid propagation, wormhole morphology, and PV_bt_. Therefore, the PV_bt_ value obtained for Sample 19 should be interpreted with caution and not as an isolated indicator of the BA formulation performance. Nevertheless, this point was retained in the data set because it was obtained under the same experimental protocol and contributes to the description of the overall BA curve. Thus, the BA results were interpreted based on the complete trend of the PV_bt_ curve, together with the wormhole morphology observed by microcomputed tomography, rather than on a single experimental point.

Additionally, the test performed at 0.3 mL/min for the BA system was classified as an incomplete/censored experiment because true breakthrough was not reached. In this case, intense dissolution near the inlet face caused loss of confinement before completion of the test. Therefore, the reported value does not represent a true PV_bt_ value, but rather partial acid consumption before mechanical failure of the confined core. This behavior is consistent with very low-flow-rate acidizing conditions, in which prolonged acid residence time near the inlet may intensify face dissolution and compromise sample integrity. For this reason, the BA-0.3 point was retained in Table 3 and in the PV_bt_ plots, but it was explicitly identified as an incomplete/censored experiment.

Therefore, although petrophysical variability exists among the samples, the results allow a consistent comparative evaluation of the studied acid systems. The corresponding PV_bt_ curves, model fitting, and postacidizing analyses are presented in the following subsections.

#### PV_bt_ Curves

Figure 4 presents the PV_bt_ curves as a function of interstitial velocity and flow rate for all evaluated formulations. The results indicate that the presence of additives reduced the minimum PV_bt_ and shifted the optimal operating window toward lower interstitial velocities. This behavior suggests lower acid consumption up to breakthrough and indicates a change in the balance between transport and reaction processes.

**Figure 4:**
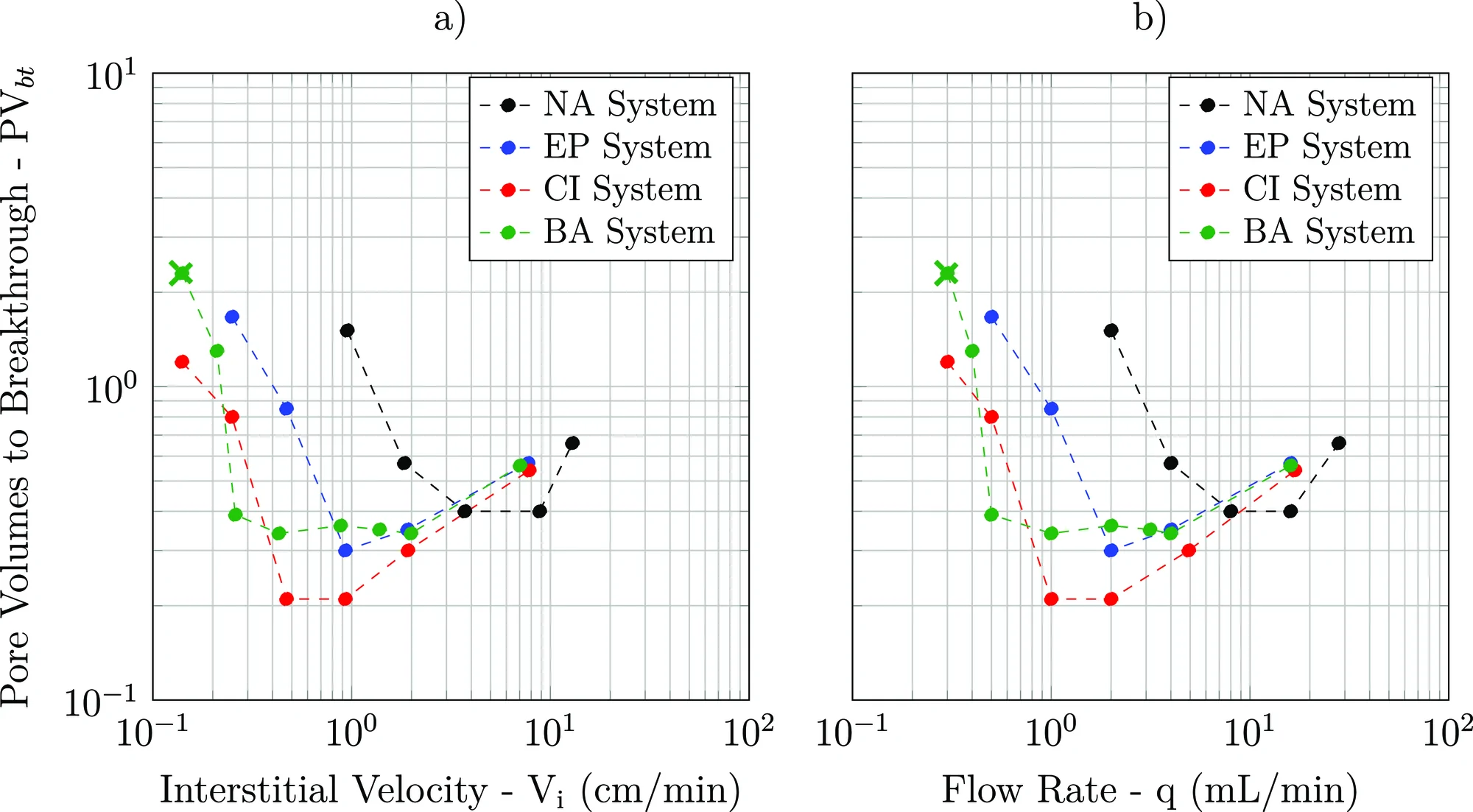
PV_bt_ as a function of (a) interstitial velocity and (b) flow rate (log–log). The point marked with an “X” in the BA system identifies the experiment performed at 0.3 mL/min, which was classified as an incomplete/censored test because true breakthrough was not reached.

Consistent with the reactor results, in which the additives reduced dissolution kinetics, the displacement of the PV_bt_ curves toward lower interstitial velocities may reflect a decrease in the effective Damköhler number. This shift suggests that lower interstitial velocities were required to restore a more favorable balance between reaction and transport, thereby promoting dominant wormhole formation under the modified kinetic conditions.^11,43,44^

Among the tested systems, the formulation containing the corrosion inhibitor (CI) exhibited the lowest PV_bt_ values, suggesting a stronger effect on acidizing performance under the evaluated conditions. In addition, the system containing both additives (BA) stands out for broadening the optimal operating region, as evidenced by a flatter minimum and a wider velocity range at low PV_bt_ values, indicating reduced sensitivity to flow rate variations.

On the other hand, the emulsion preventer system (EP) showed a moderate reduction in PV_bt_ compared to the nonadditive system (NA), indicating a smaller shift in PV_bt_ relative to the other additive-containing systems.

Based on Figure 4 and Table 3, the system without additives (NA), the lowest PV_bt_ value was 0.40, observed at flow rates of 8 and 16 mL/min, corresponding to interstitial velocities of 3.71 and 8.83 cm/min, respectively. In contrast, the acid containing the emulsion preventer (EP) exhibited lower PV_bt_ values at lower flow rates and interstitial velocities, with an optimum value of 0.30 PV at a flow rate of 2 mL/min and an interstitial velocity of 0.93 cm/min. From the experimental results, it is possible to estimate an approximate 25% reduction in acid consumption, represented by the downward shift of the PV_bt_ curve for the EP system relative to the nonadditive system (NA).

Similarly, the presence of the corrosion inhibitor (CI) resulted in a more pronounced reduction in both PV_bt_ values and interstitial velocities, shifting the optimal condition to even lower values. By correlating the curves with the experimental results presented in Table 3, the optimal condition for the CI corresponds to the minimum PV_bt_ value of 0.21, observed at flow rates of 1 and 2 mL/min and interstitial velocities of 0.47 and 0.93 cm/min, respectively.

The CI-containing system exhibited an approximate 48% reduction in acid consumption, along with a shift in the optimal condition toward interstitial velocities about 4 to 8 times lower than those observed for the nonadditive system. Additionally, it showed a 30% reduction in acid consumption compared to the EP system under similar flow rate and interstitial velocity conditions.

Under the conditions investigated, the results suggest that CI had the strongest effect on acidizing performance, as it produced the most pronounced reduction in PV_bt_ and was also associated with the greatest delay in reaction kinetics among the tested formulations.

As observed for the individual additives, the formulation containing both additives also shifted the PV_bt_ curve. Compared to the nonadditive system, a moderate 15% reduction in the minimum value was observed. Beyond the reduction in minimum PV_bt_, the expansion of the optimal operating range is an important feature of this system, as the similarly low PV_bt_ values observed at injection rates of 1, 2, and 4 mL/min may offer operational advantages by reducing sensitivity to flow-rate variations and potentially lowering the risk of unintended fracture initiation.

#### Buijse–Glasbergen Model Fitting

Figure 5 presents the experimental PV_bt_ data and the corresponding Buijse–Glasbergen (B&G) model fits for the NA, EP, CI, and BA systems.

**Figure 5:**
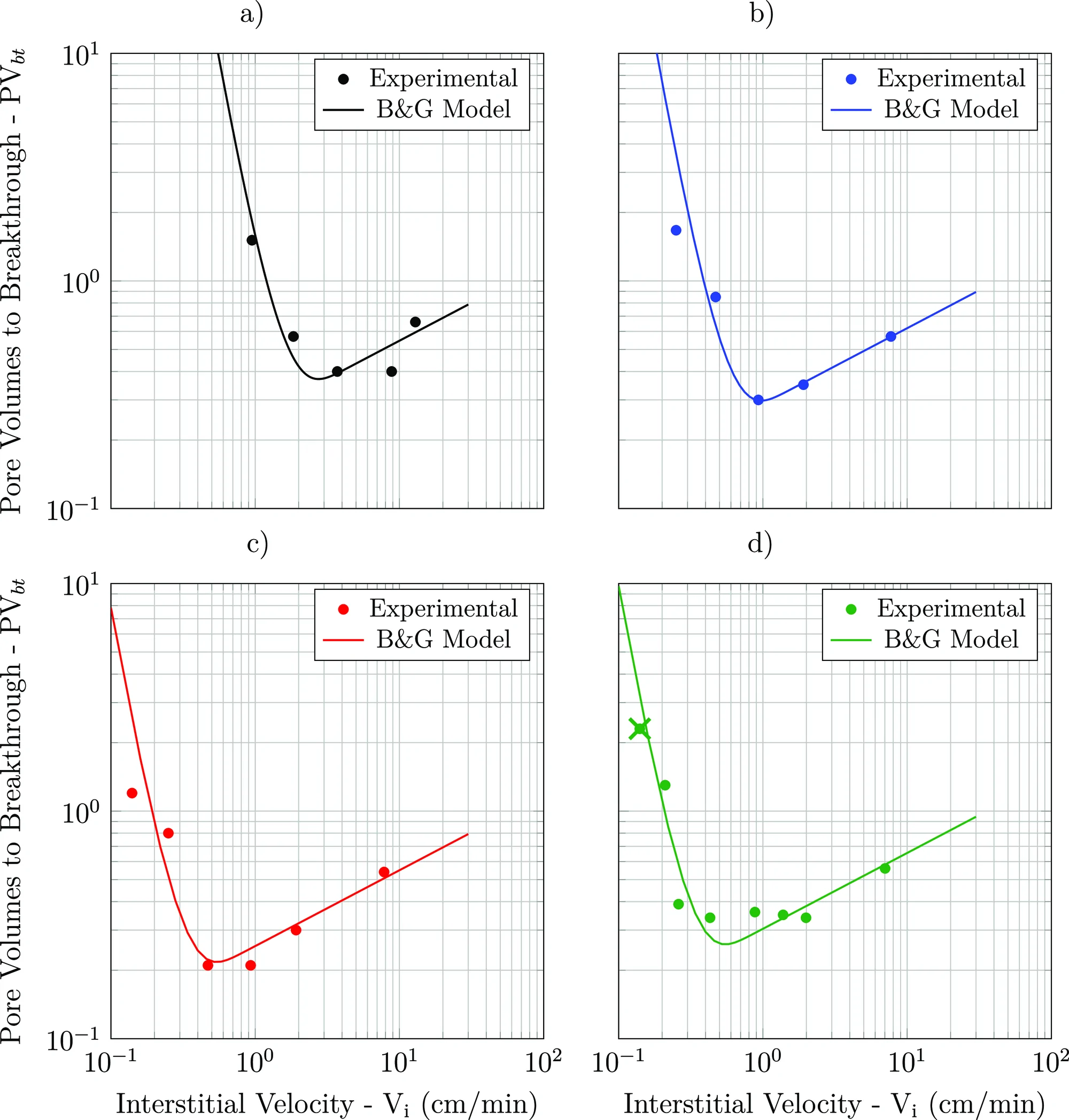
PV_bt_ as a function of interstitial velocity (log–log) for the systems: a) No Additives (NA), b) Emulsion Preventer (EP), c) Corrosion Inhibitor (CI), and d) Both Additives (BA), comparing experimental data with the Buijse–Glasbergen model. The point marked with an “X” in the BA system identifies the experiment performed at 0.3 mL/min, which was classified as an incomplete/censored test because true breakthrough was not reached.

In all cases, the model provided satisfactory agreement with the experimental results and reproduced the overall trend of the experimental curves, including the steeper PV_bt_ variation at low interstitial velocities, the shift in the optimum region, and the more gradual trend at higher interstitial velocities.

These results support the use of the Buijse–Glasbergen approach as a useful framework for interpreting the influence of the additives on carbonate acidizing performance under the conditions evaluated.^44^

Figure 6 presents the experimental PV_bt_ data and the corresponding Buijse–Glasbergen model predictions in grouped form, allowing a direct comparison among the acid systems.

**Figure 6:**
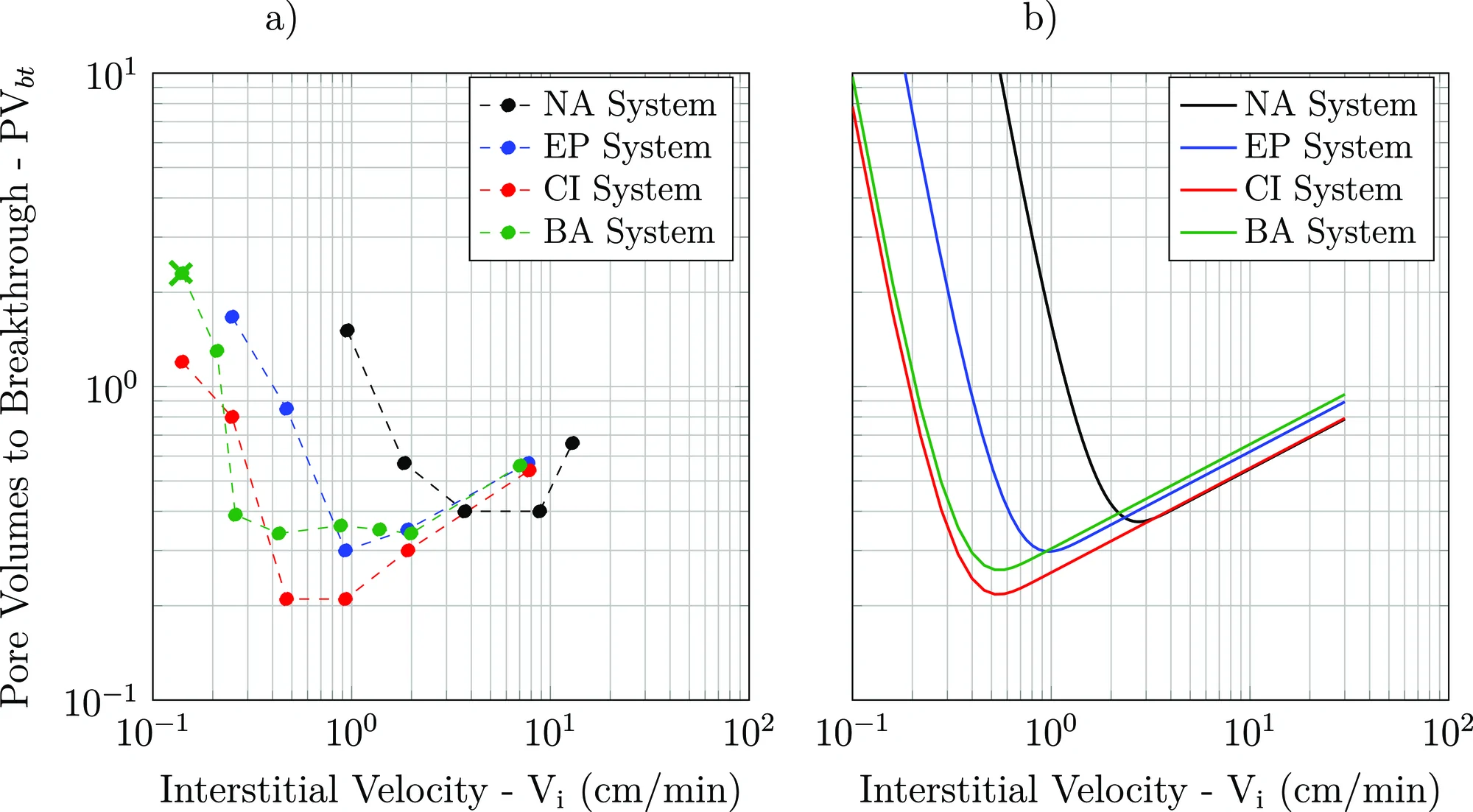
PV_bt_ vs interstitial velocity (log–log): a) Experimental data and b) Buijse–Glasbergen model predictions. The point marked with an “X” in the BA system identifies the experiment performed at 0.3 mL/min, which was classified as an incomplete/censored test because true breakthrough was not reached.

The NA system showed the least favorable behavior, with the minimum PV_bt_ occurring at a much higher interstitial velocity and with a higher PV_bt_ value. The EP system showed an intermediate response, whereas the CI and BA systems exhibited lower minima at lower interstitial velocities, indicating more favorable conditions for dominant wormhole formation and lower acid consumption up to breakthrough. At high interstitial velocities, the curves show a similar trend, suggesting that the effect of the additives becomes less pronounced in this region.

Table 4 provides a complementary view of these trends through the fitted model parameters and the predicted optimal conditions.

**Table 4:** Buijse–Glasbergen Model Parameters (*W*_eff_, *W*_B_) and Estimated Optimal Conditions (*V*_i‑opt_ and PV_bt‑opt_) for Each Acid System

Acid System	*W*_eff_ (cm/min)^1/3^	*W*_B_ (cm/min)^−2^	*V*_i‑opt_ (cm/min)	PV_bt‑opt_
NA	3.95	0.51	2.75	0.37
EP	3.48	4.07	0.90	0.30
CI	3.92	13.15	0.54	0.22
BA	3.29	12.84	0.55	0.26

The differences in dissolution efficiency and wormhole formation among the four acid systems can be assessed through the *W*_eff_ and *W*_B_ parameters, as well as the resulting values of *V*_i‑opt_ and PV_bt‑opt_. The values of *W*_eff_ show how each system vertically shifts the PV_bt_ curve: higher *W*_eff_ corresponds to an overall decrease in PV_bt_ across all velocities without changing the curve’s shape or the horizontal position of the optimum point.^21^

In this context, the NA and CI systems exhibit the highest W_eff_ values: 3.95 (cm/min)^1/3^ and 3.92 (cm/min)^1/3^). It indicates a natural tendency toward lower PV_bt_ throughout the entire curve. This vertical shift is reinforced by the close overlap of the NA and CI curves at *V*_i_ values above the *V*_i‑opt_ of the NA system, where both systems exhibit very similar PV_bt_ values. In contrast, BA shows the lowest *W*_eff_ 3.29 (cm/min)^1/3^, which is consistent with its comparatively higher PV_bt_ response despite its favorable wormholing behavior at low velocities.

The *W*_B_ values complement this interpretation. When *W*_B_ increases, the optimal region of the PV_bt_ curve shifts diagonally toward the lower-left portion of the plot, resulting in lower *V*_i‑opt_ and PV_bt‑opt_ values. In contrast, when *W*_B_ is smaller, this optimal region moves toward the upper-right diagonal. Importantly, the high interstitial velocity region of the curve remains unchanged regardless of the *W*_B_ magnitude.^21^

This trend is evident in the CI and BA systems, which have the highest W_B_ values 13.15 (cm/min)^−2^ and 12.84 (cm/min)^−2^ and consequently exhibit the lowest *V*_i‑opt_ values, both around 0.54–0.55 cm/min. Thus, these systems require low injection velocities to reach their optimal region. In contrast, the NA system, with the lowest *W*_B_ – 0.51 (cm/min)^−2^, places its optimum at a much higher velocity (2.75 cm/min), which explains why its PV_bt‑opt_ (0.37) is the highest in the Table 4, even though it exhibits the most favorable *W*_eff_ value.

When the parameters are evaluated together, the CI system stands out as the most efficient among the four acid systems. Its high W_eff_ and W_B_ act synergistically, resulting in the lowest PV_bt_-opt (0.22) and most favorable *V*_i‑opt_. BA also benefits from a high *W*_B_ and reaches a similarly low *V*_i‑opt_, though its lower *W*_eff_ prevents it from achieving a PV_bt‑opt_ as low as CI. EP falls in an intermediate position: its *W*_eff_ and *W*_B_ values shift both the curve and the optimum toward more efficient conditions, but not to the same extent as CI. Finally, NA requires the highest injection velocity to reach its optimal behavior and still delivers the largest PV_bt‑opt_, showing that the relatively high *W*_eff_ is not sufficient to offset the effect of the low *W*_B_ value.

#### Rock Surface Analysis after Acid Flow

The inlet-face dissolution patterns obtained after acid injection are presented in Figure 7. Because no relevant visual differences were observed at the outlet faces, the discussion focuses on the inlet region, where the first interaction between the acid and the carbonate matrix.

**Figure 7:**
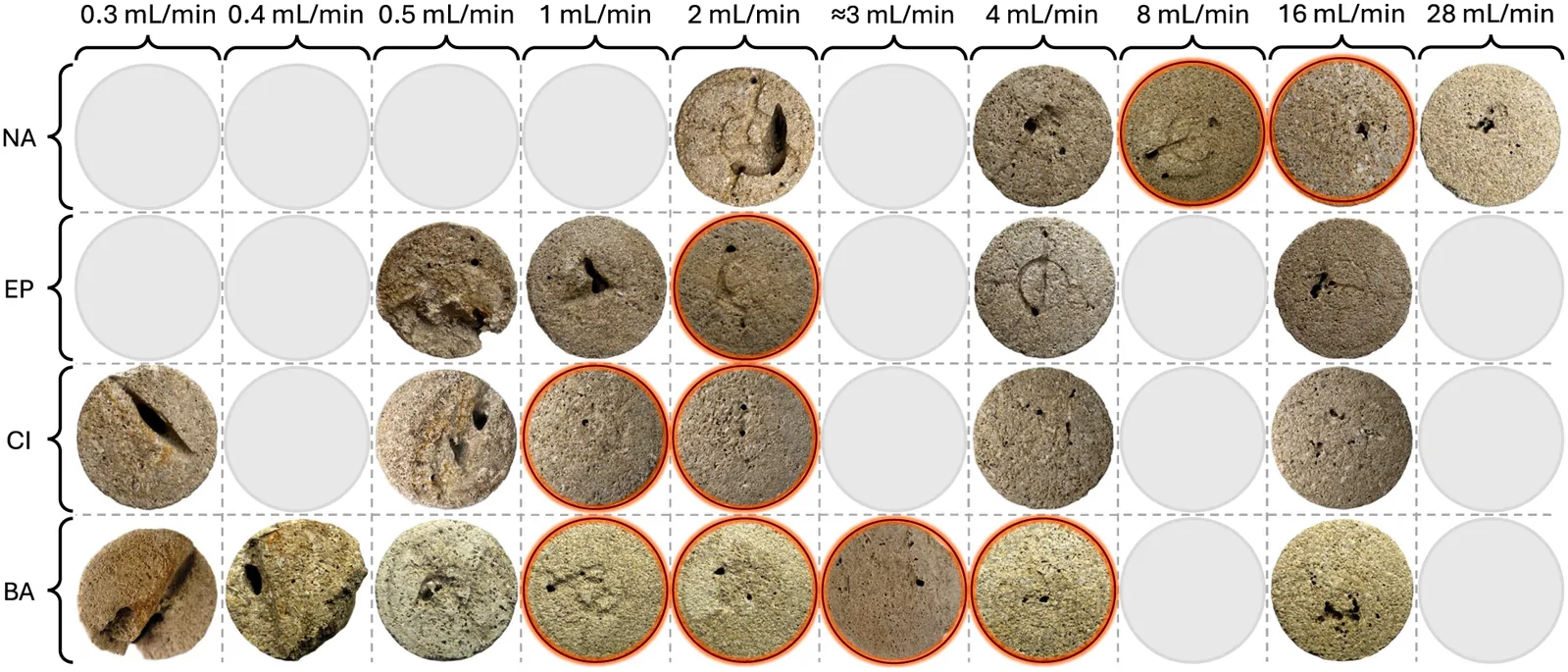
Dissolution patterns of the inlet face for different flow rates and acid systems (optimal experimental conditions are highlighted in red).

For analysis purposes, the experimental conditions in this subsection are classified, for each acid system, into low, intermediate, and high injection-rate conditions. In this context, the inlet faces highlighted in red correspond to the intermediate experimental conditions associated with the lowest PV_bt_ values, thereby representing the optimal point or region for each acid system. Accordingly, flow rates lower than those associated with this condition are classified as low, whereas higher flow rates are classified as high.

In general, the inlet face images show that the acid systems produced distinct dissolution patterns depending on both the acid formulation and the injection condition, which is consistent with the interpretation of carbonate acidizing regimes reported in the literature.^8,11,30^ At low injection rates, the reduced fluid velocity promotes longer acid–rock contact times, limiting acid transport into the core and intensifying face dissolution at the inlet, along with surface pore enlargement. Even under these conditions, however, the injection rate remains sufficient to sustain wormhole propagation through the sample and eventually reach breakthrough.

As the injection rate approaches the optimal region, dissolution becomes more localized under these intermediate conditions, indicating that the reactive fluid interacts with the rock matrix less dispersively at the inlet face. This behavior suggests a more efficient use of the acid, as it promotes localized dissolution points that favor wormhole propagation, consistent with the observed reduction in PV_bt_ and later confirmed by microtomography.

At higher injection rates, an increase in the dispersion of acid attack at the inlet face is observed, with multiple dissolution initiation points. This distribution of the reactive flow corroborates the increase in acid consumption.

In the NA system, the inlet-face images suggest a stronger tendency toward acid consumption near the rock surface at conditions below the optimal region. This is consistent with the batch reactor results, which showed the fastest dissolution kinetics for the additive-free acid, and with the higher PV_bt_ values observed for this formulation. The more reactive character of this system helps explain the more intense face dissolution and surface pore enlargement observed at the inlet. In addition, the optimal conditions for the NA system occurred at higher flow rates and interstitial velocities than those of the additive-containing systems, reinforcing the stronger acid–rock interaction at the inlet.

In contrast, the additive-containing systems generally exhibited more discrete and localized entry features under intermediate flow conditions, which corroborate the literature.^21,30,45^ This behavior is consistent with the batch reactor results, which showed that the additives significantly increased the reaction time. Under these conditions, the acid can penetrate further into the rock before being consumed, even at lower flow rates, favoring deeper wormhole propagation.

#### Microcomputed Tomography Analysis

The internal dissolution patterns generated during the acidizing experiments were analyzed by microcomputed tomography, as shown in Figures 8 to 12. This technique provides a three-dimensional visualization of wormhole structures, enabling direct assessment of how acid formulations and injection conditions influence the development, connectivity, and morphology of the dissolution channels.

**Figure 8:**
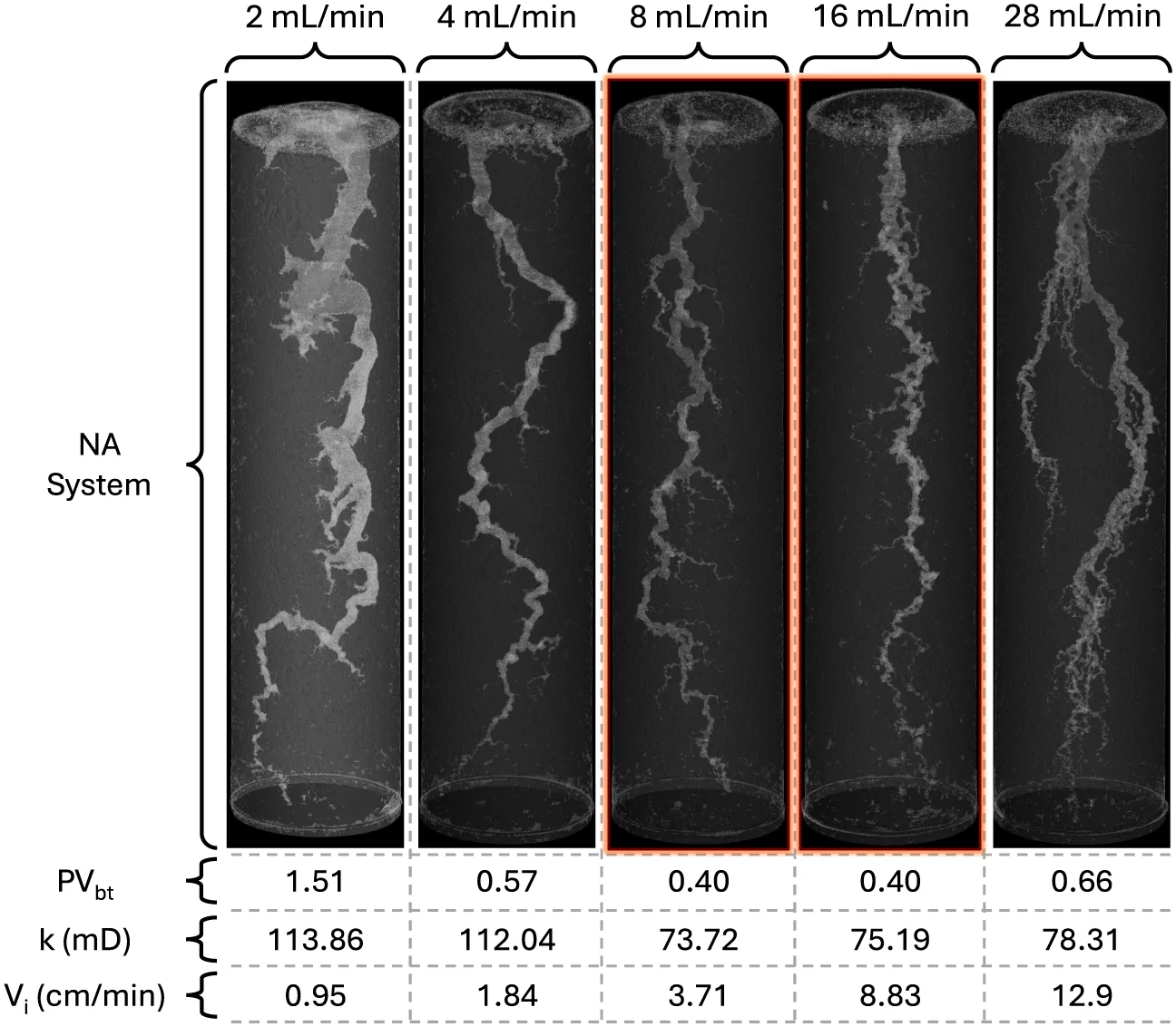
μCT images of wormhole structures generated during acidizing experiments using the NA system (15 wt % HCl without additives) at different injection rates. The images are organized according to increasing flow rate, with the corresponding PV_bt_, permeability, and interstitial velocity values indicated below each sample. The conditions highlighted in red correspond to the minimum PV_bt_ values, representing the most efficient wormhole propagation conditions.

Figure 8 presents the results for the NA system under different injection rates. At low flow conditions (2 mL/min), the dissolution pattern shows a wider structure with channel widening, similar to the conical wormhole pattern described in the literature, characterized by the predominance of dissolution near the entrance face.^11^ As the injection rate increases, a transition from the conical to the optimal regime is observed at 4 mL/min, while the optimal condition for this system is achieved at 8 and 16 mL/min, where more defined and dominant wormholes are formed, indicating more efficient acid propagation and lower consumption. At higher rates (28 mL/min), the pattern becomes more elongated but also more branched, indicating increased channel competition and less efficient acid utilization. This behavior is consistent with the classical transition from face dissolution to dominant wormhole formation and, subsequently, to branched regimes at higher flow rates, as reported in carbonate acidizing studies.^10,46,47^

Figure 9 shows the dissolution patterns for the EP system. Compared with the NA system, the presence of the emulsion preventer promotes more defined and continuous channels, particularly at intermediate injection rates (2–4 mL/min). The wormholes exhibit a less diffuse dominant pattern, indicating a more favorable balance between reaction and mass transfer. At very low flow rates, dissolution remains relatively uniform, whereas at higher rates (16 mL/min), branching becomes more evident. Changes in wormhole patterns under the same flow conditions support the interpretation that the additive alters interfacial behavior and acid distribution within the porous medium.

**Figure 9:**
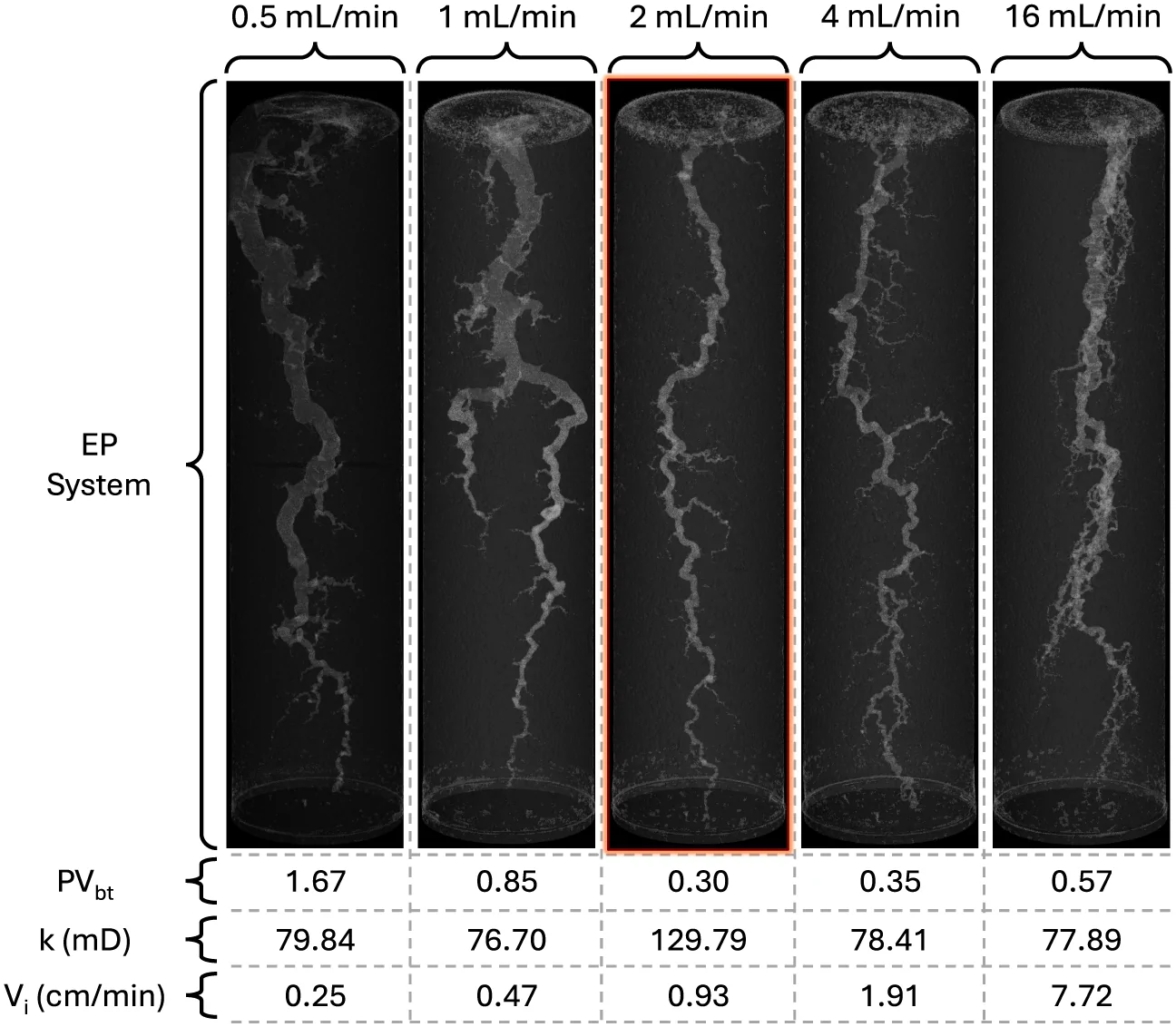
μCT images of wormhole structures generated during acidizing experiments using the EP system (15 wt % HCl with emulsion preventer) at different injection rates. The images are organized according to increasing flow rate, with the corresponding PV_bt_, permeability, and interstitial velocity values indicated below each sample. The conditions highlighted in red correspond to the minimum PV_bt_ values, representing the most efficient wormhole propagation conditions.

For the CI system (Figure 10), a shift in wormhole behavior is observed. A dominant wormhole pattern develops at relatively low interstitial velocities (0.47–0.93 cm/min), corresponding to lower injection flow rates (1–2 mL/min) compared with NA system. The channels exhibit greater penetration and reduced lateral branching compared with the NA and EP systems, which is consistent with the lower PV_bt_ values observed experimentally.

**Figure 10:**
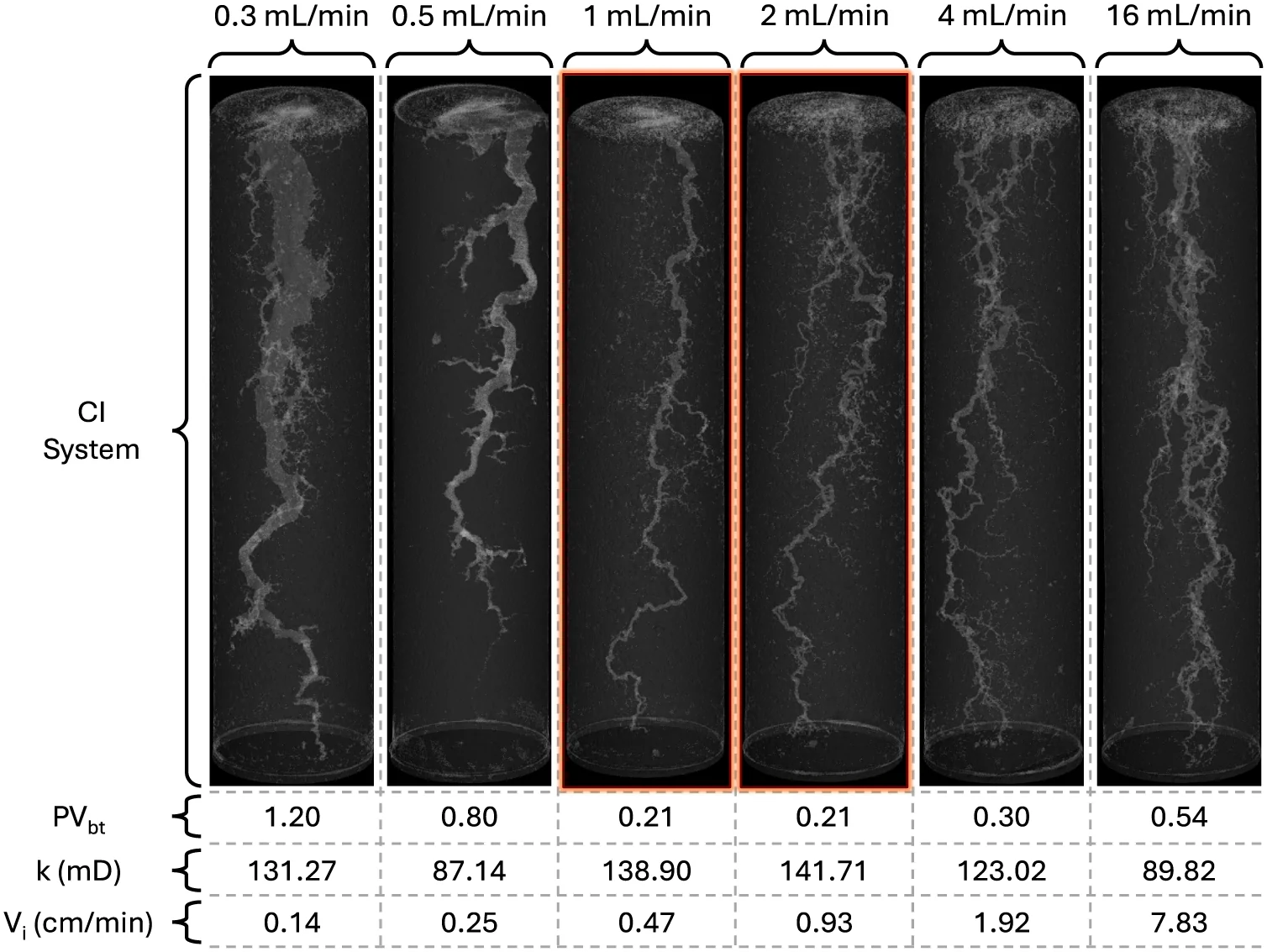
μCT images of wormhole structures generated during acidizing experiments using the CI system (15 wt % HCl with corrosion inhibitor) at different injection rates. The images are organized according to increasing flow rate, with the corresponding PV_bt_, permeability, and interstitial velocity values indicated below each sample. The conditions highlighted in red correspond to the minimum PV_bt_ values, representing the most efficient wormhole propagation conditions.

Under low flow conditions of the CI system (0.3 and 0.5 mL/min), the formation of a conical channel is observed, consistent with the images of the face with high surface dissolution for the development of channel propagation. At higher injection rates, the structures become more complex and branched. In the CI system, branching can already be observed from 2 mL/min onward, whereas in the EP and NA systems this behavior becomes evident only between 4 and 16 mL/min and between 16 and 28 mL/min, respectively. This earlier appearance of branching in the CI system may be associated with the lower reaction rate of this formulation compared with the other acid systems.

The BA system (Figure 11) combines the effects of both additives and exhibits intermediate behavior. At intermediate flow rates (1–4 mL/min), a transition toward a dominant pattern is observed.

**Figure 11:**
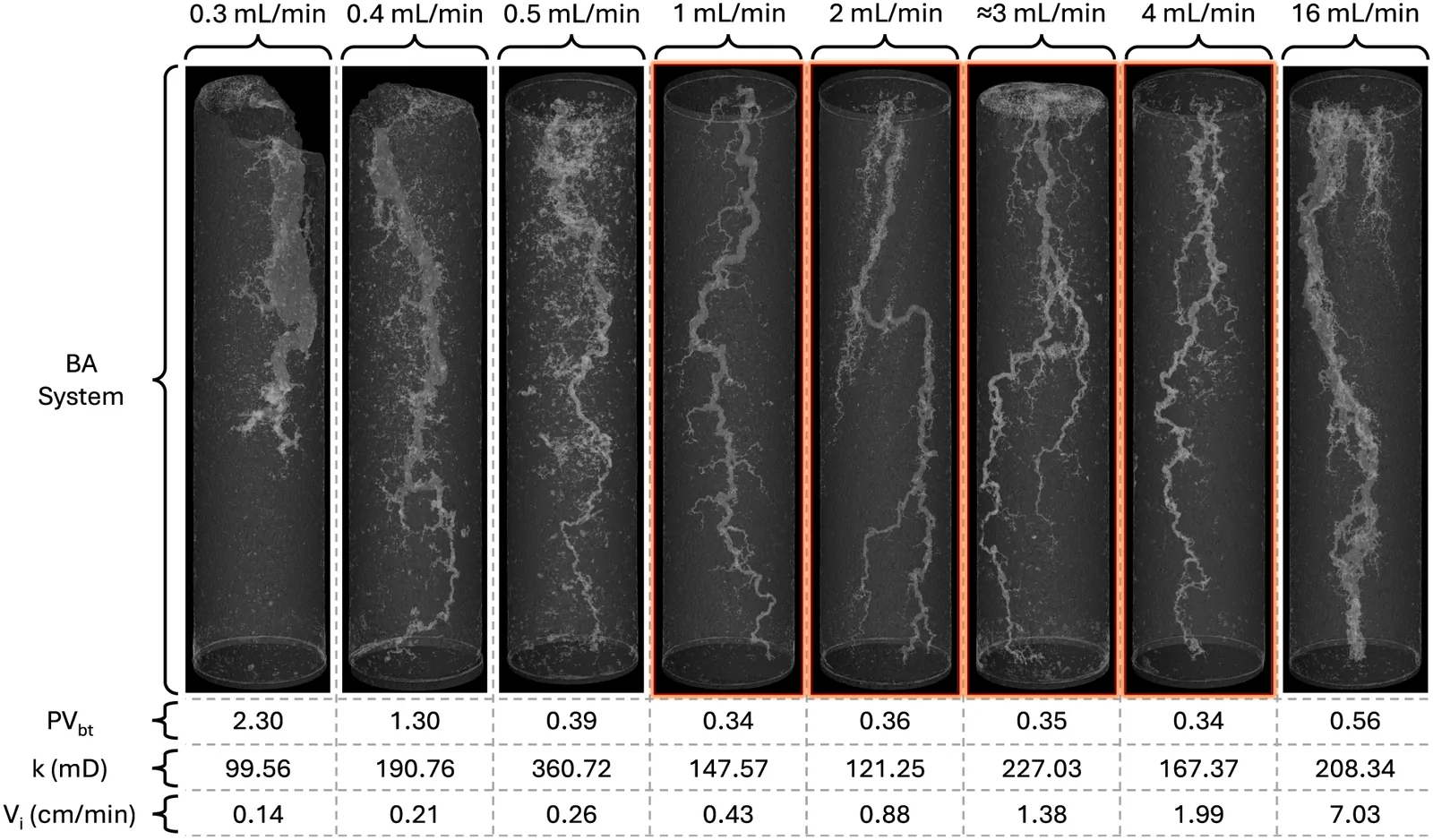
μCT images of wormhole structures generated during acidizing experiments using the BA system (15 wt % HCl with emulsion preventer and corrosion inhibitor) at different injection rates. The images are organized according to increasing flow rate, with the corresponding PV_bt_, permeability, and interstitial velocity values indicated below each sample. The conditions highlighted in red correspond to the minimum PV_bt_ values, representing the most efficient wormhole propagation conditions. The experiment performed at 0.3 mL/min in the BA system was classified as an incomplete/censored test because true breakthrough was not reached.

Compared with the CI system, the BA system showed dominant wormholes over a wider flow rate interval. In this range, the wormholes formed with BA tend to have larger diameters than those observed for CI, which is consistent with the higher PV_bt_ values of the BA system. In addition, branching in the CI system is already observed at 2 mL/min, whereas in the BA system it becomes evident only from 3 mL/min and is more pronounced at 4 mL/min. As the injection rate increases, branching becomes more pronounced, indicating reduced acid utilization efficiency.

In contrast, at low injection rates, dissolution is concentrated near the inlet region, indicating early acid consumption. This behavior is supported by the test at 0.3 mL/min, in which intense dissolution at the inlet face led to a loss of confinement, preventing test completion. Microtomography analysis further confirms that, under these conditions, additional acid volumes would be required to achieve full penetration of the sample.

A direct comparison between the four systems under the same injection condition (2 mL/min) is presented in Figure 12. The NA system shows a wider channel, similar to a conical wormhole pattern, while the EP system presents a more localized wormhole. The CI system exhibits the most efficient wormhole structure, characterized by a single dominant channel that is narrower than that observed in the EP system. The BA system also shows a continuous wormhole, though with slightly more branching than in CI. These observations agree with the PV_bt_ analysis and the Buijse–Glasbergen model results,^44^ confirming that the additives significantly influence the efficiency of wormhole formation.

**Figure 12:**
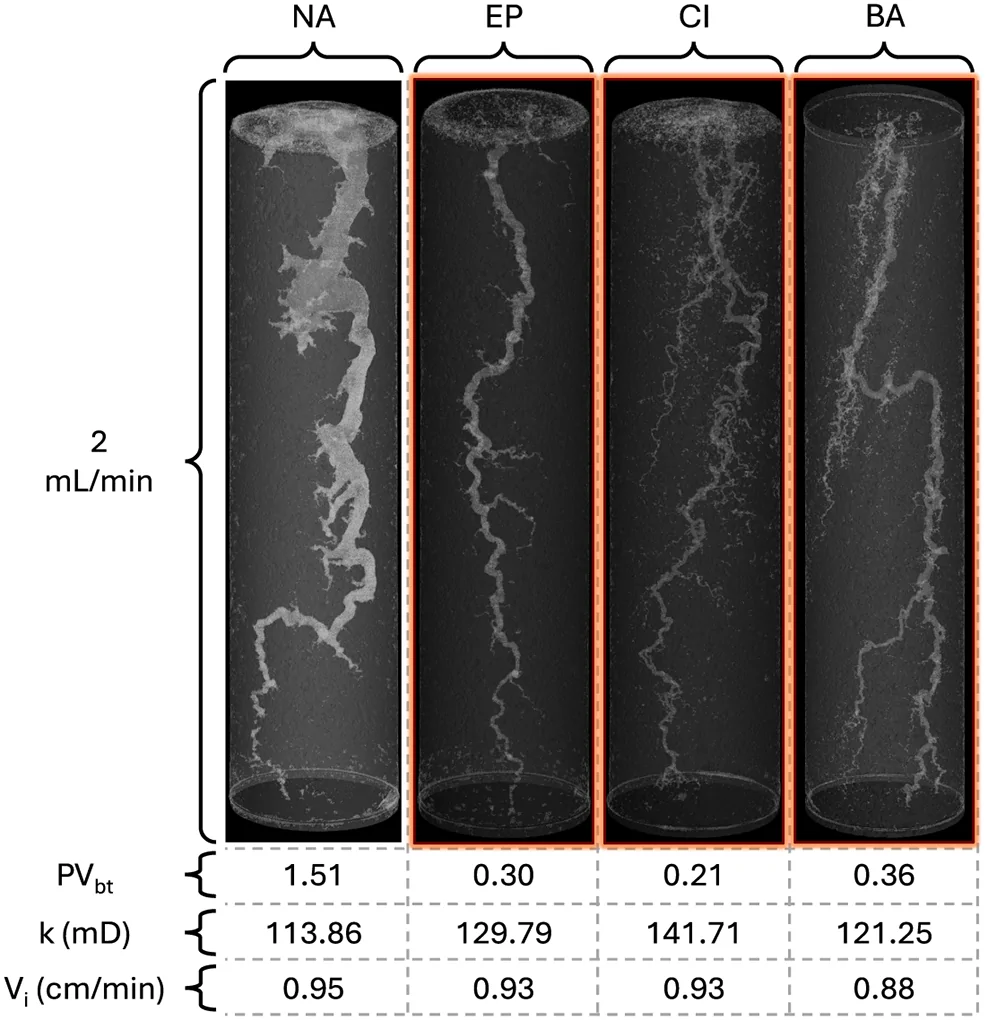
Comparison of wormhole structures obtained by μCT for the NA, EP, CI, and BA systems at the same injection rate (2 mL/min). The corresponding PV_bt_, permeability, and interstitial velocity values are indicated below each sample. The red-highlighted images represent the experimental conditions associated with the most efficient wormhole propagation for each system.

Microtomography results indicate that, even under comparable experimental conditions of flow rate, temperature, confinement, and backpressure, different acid systems produced distinct dissolution patterns, suggesting that the additives influenced the acidizing process. Consistent with the reactor tests, in which the presence of additives delayed rock dissolution, changes in acid consumption and fluid propagation behavior were also observed in porous media. Together, these effects were reflected in the channel configurations observed by microtomography, providing additional insight into the operating conditions associated with the development of a well-defined dominant channel.

Overall, the results suggest that the additives affected wormhole development and PV_bt_ behavior through changes in the balance between reaction and transport, favoring dominant channel formation at lower acid consumption and under lower flow-rate and interstitial-velocity conditions.

## Conclusion

This study integrates physicochemical characterization, batch reactor kinetics, and core flooding experiments in porous media to assess the impact of additives on carbonate acidizing performance. By combining dissolution rate analysis, PV_bt_ curves, surface observations, and microtomography, it was possible to evaluate how the corrosion inhibitor (CI), emulsion preventer (EP), and their combined formulation (BA) influence both reaction kinetics and wormhole development.

The results show that the addition of additives does not significantly affect density and viscosity compared with additive-free HCl. In contrast, surface tension was the most sensitive physicochemical parameter, showing a pronounced reduction in the presence of surfactant-based additives.

Dissolution kinetics were strongly influenced by fluid composition. The presence of additives resulted in a marked retardation effect, particularly with the corrosion inhibitor, which increased the total dissolution time by up to 30 times. The emulsion preventer and the combined system showed more moderate effects, increasing dissolution time by approximately 3–4.5 times and 18–20 times, respectively.

This reduction in reaction rate directly impacted reactive flow efficiency. The CI system reduced the minimum PV_bt_ by approximately 48%, while EP and BA reduced it by about 25% and 15%, respectively, compared with additive-free HCl. Under the investigated conditions, the lower reactivity induced by the additives led to more efficient acid utilization, thereby reducing the volume required to achieve breakthrough.

The decrease in reactivity shifted the optimal acidizing condition toward lower interstitial velocities, as evidenced by the displacement of the PV_bt_ curves. The combined system (BA) showed a moderate reduction in PV_bt_ and a broader low-PV_bt_ region under the investigated laboratory conditions, with values between 0.34 and 0.36 PV for flow rates from 1 to 4 mL/min.

Surface analysis revealed that low injection rates promoted uniform face dissolution, while optimal conditions minimized face dissolution and favored localized channel formation. At higher flow rates, the number of dissolution initiation points increased, indicating channel competition. The presence of CI reduced face dissolution under low flow conditions, reinforcing its retardation effect.

Post flow tomography analysis corroborated these observations, clearly identifying the transition between dissolution patterns. At low flow rates, homogeneous dissolution near the inlet dominated; under optimal conditions, a single dominant wormhole was formed; and at high flow rates, branched structures with multiple competing channels were observed. Furthermore, the presence of additives modified the wormhole geometry, leading to the formation of dominant channels at lower injection rates, particularly in the CI system.

The systems containing additives also exhibited more axially continuous wormholes under optimal conditions, confirming the consistency between reduced reactivity, improved channel efficiency, and lower PV_bt_. For all formulations, the PV_bt‑opt_ values were associated with dominant wormhole formation, whereas higher acid consumption was associated with face dissolution or excessive branching.

Overall, the results demonstrate that matrix acidizing efficiency is not governed solely by injection rate, but by the balance between fluid reactivity and transport. Additives play a key role in modifying this balance by altering interfacial properties and reaction kinetics, thereby altering the conditions for wormhole development. These findings highlight the importance of evaluating acid formulations on a case-by-case basis, as relatively small compositional changes can lead to significant differences in dissolution behavior, wormhole morphology, and acid utilization efficiency.
